# Reframing Food is Medicine as syndemic care: a multidimensional approach to food insecurity

**DOI:** 10.3389/fpubh.2026.1776957

**Published:** 2026-02-17

**Authors:** Caroline Elizabeth Owens, David Himmelgreen

**Affiliations:** 1 Department of Anthropology, University of North Carolina Wilmington, Wilmington, NC, United States; 2 Department of Anthropology, University of South Florida, Tampa, FL, United States

**Keywords:** chronic disease, Food is Medicine, food security, nutrition, syndemic approach

## Abstract

Food insecurity and chronic disease are interlinked through systemic disparities in the United States. Building on anthropological and public health scholarship, we argue that syndemic theory offers a valuable framework for analyzing these entangled conditions. A syndemic lens highlights overlapping biological, behavioral, and social mechanisms. Such as chronic stress, inflammation, and coping strategies, that exacerbate disease outcomes in populations experiencing food insecurity, while also pointing toward testable pathways for intervention. In this essay, we explore the potential promise of Food is Medicine (FIM), which includes clinically embedded approaches like medically tailored meals and produce prescriptions, as a form of syndemic care, targeting the co-occurrence of social and health vulnerabilities and risks. Emerging evidence suggests that FIM programs can improve food security, glycemic control, and support mental health. However, recent federal policy changes, including proposed cuts to Medicaid and the Supplemental Nutrition Assistance Program, threaten to undermine these interventions and syndemic care more broadly. Only by addressing both proximate needs and root causes can syndemic care disrupt entrenched cycles of disadvantage and advance population-level health and health equity.

## Introduction

Over 75% of all adults in the United States live with one or more chronic conditions, many of which are diet-sensitive chronic diseases or conditions. Co- and multimorbidity have become increasingly prevalent, with one in two adults managing multiple, overlapping chronic health conditions (1). These clustered health conditions are patterned by structural and social factors, including county-level economic status (2), systemic discrimination (3, 4), and insecurity related to basic resources like housing, healthcare, and food (5, 6). As a result, food insecurity contributes to the disproportionate prevalence of diet-sensitive diseases among Black or African American, Hispanic/Latinx, American Indian and Alaska Native communities (7), as well as among individuals with lower incomes (8). The recent rise of food insecurity in the United States (9), coupled with policy changes introduced by the One Big Beautiful Bill Act (H.R.1-119th Congress), including cuts to Medicaid and food assistance programs, threatens to deepen existing health disparities, worsen the burden of diet-sensitive chronic disease, and lead to 16,000 extra deaths annually (10).

Food insecurity has wide-ranging consequences for health and wellbeing (5). One well-documented example is its relationship to type 2 diabetes. Compared to people with food security, those experiencing food insecurity are two to three times more likely to have type 2 diabetes and significantly more likely to have poor glycemic control, lower diabetes-specific self-efficacy, and diabetes distress (11–13). In addition to its effects on physical health, food insecurity is robustly associated with adverse mental health conditions, including depression and anxiety (14), which are common comorbidities of diet-related cardiometabolic conditions like type 2 diabetes (15).

## Syndemics: the interplay of disease and social disparities

The frequently overlapping constellation of social and economic marginalization, food insecurity, and diet-related chronic diseases has led some scholars to conceptualize these conditions as syndemic phenomena (16, 17). Anthropologist Merrill Singer developed the concept of syndemics, which broadly describes the clustering and synergistic interactions of diseases or health conditions shaped by and exacerbating existing social inequalities (18). Importantly, not all comorbidities meet the criteria for syndemics. Emily Mendenhall identifies three defining features: (1) the co-occurrence of two or more diseases within a specific population; (2) social and contextual factors that are co-constructed and contribute to this co-occurrence; and (3) interactions between diseases that intensify their impact, thereby compounding impacts among affected populations (19).

Building on this framework, Himmelgreen and colleagues argue that food insecurity and diet-related chronic disease constitute a syndemic (17). Himmelgreen et al. suggest that dietary intake, coping strategies, and the biological effects of chronic stress—including dysregulation in cortisol, increased inflammation and the accumulation of visceral fat—interact with the effects of poverty to perpetuate this syndemic. Supporting these proposed biological mechanisms, food insecurity has been associated with biomarkers of inflammation in the US adult population (20). Moreover, both C-reactive protein, an inflammatory biomarker, and cortisol, a stress hormone, have been shown to partially mediate the relationship between household food insecurity and insulin resistance among Latinos with type 2 diabetes (21).

**Figure 1:**
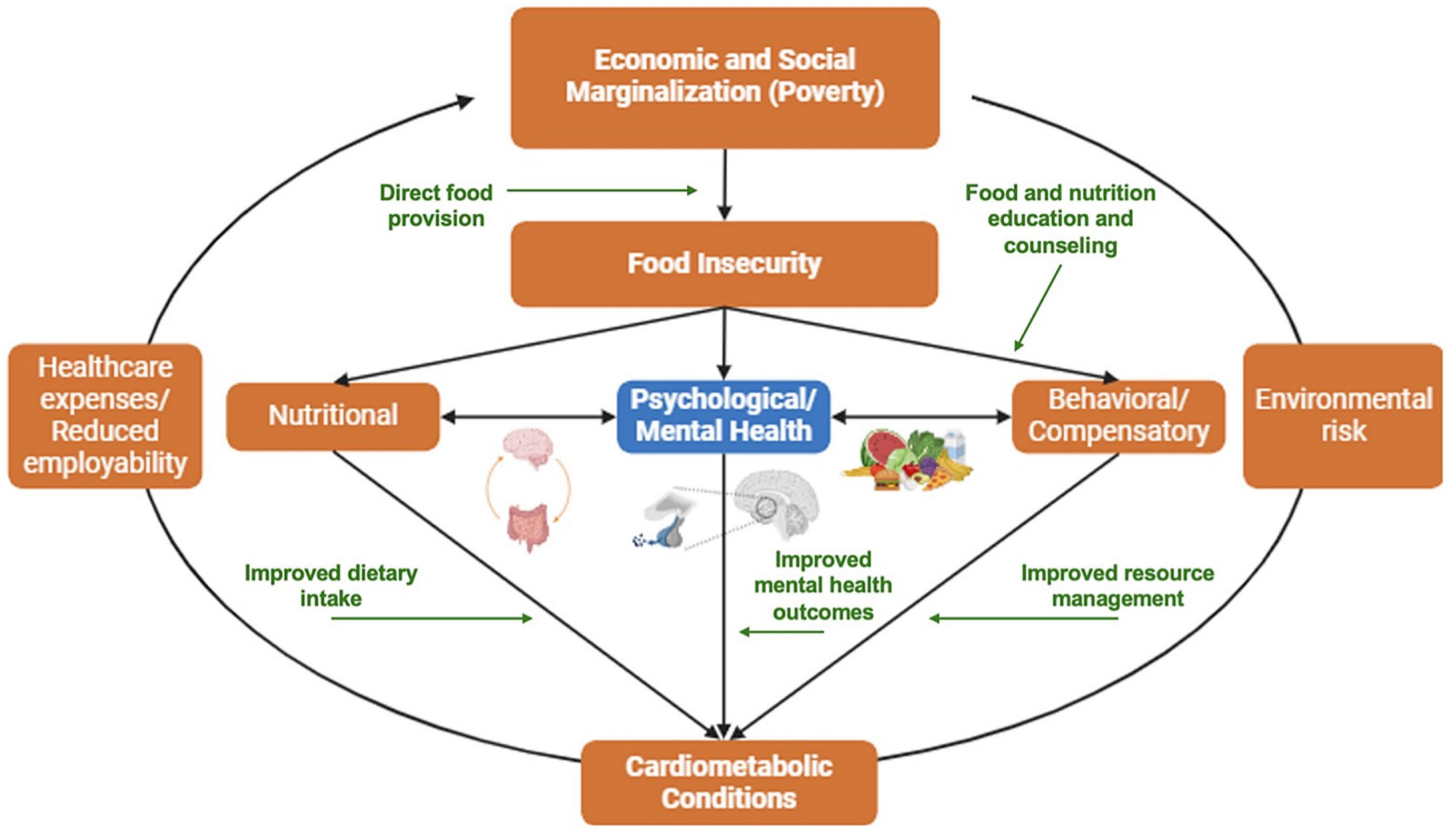
Proposed syndemic of food insecurity, mental health, and cardiometabolic outcomes, and pathways of Food is Medicine (FIM) impact in green.

Beyond biological mechanisms, individuals experiencing food insecurity also report coping strategies, such as disordered eating and medication scrimping and non-adherence, that can worsen metabolic outcomes and contribute to emotional distress (22). These findings point to intersecting biological, behavioral, and social pathways, as illustrated in Figure 1, and suggest that interventions grounded in a syndemic framework may be uniquely positioned to disrupt vicious cycles of food insecurity and poor health. However, as Berkowitz, Seligman, and Mozaffarian caution (23), strategies that improve income or food security may enhance certain aspects of health without necessarily improving dietary quality or nutrition security. Similarly, effort focused narrowly on food security or diet may fail to address the broader, systemic drivers of resource insecurity. These considerations underscore the need for holistic, multi-level interventions that address structural drivers of the proposed syndemic.

## Syndemic care: from theory to practice

While syndemic theory has gained traction in public health research as a framework for understanding the interaction of health conditions and structural inequities, its translation into equity-oriented clinical and public health interventions remains nascent. Nonetheless, syndemic thinking offers a promising lens for reimagining care delivery, particularly by emphasizing how chronic stress and systemic disadvantage shape health outcomes and engagement with treatment.

As conceptualized by Dixon and Mendenhall, “syndemic care” applies this framework to clinical practice and policy by emphasizing treatment approaches that addresses co-occurring conditions holistically rather than in isolation (24). Syndemic care expands on integrative or holistic models of medicine by addressing not only shared biological etiologies of disease but the social contexts by which they manifest. This approach moves beyond personal health optimization to encompass the social and environmental dimensions of health. In practice, this means designing interventions that are integrated, contextually aware, and responsive to disadvantage, marginalization, and disenfranchisement. Syndemic care thus requires care teams to understand the lived experiences of patients. In doing so, syndemic care offers a deeper response to the root causes of health than models primarily focused on individual agency or behavior. Existing social determinants of health screening and referral processes are a vital initial step toward moving beyond biomedical reductionism. Realizing the full potential of syndemic care requires clinical systems to connect patients with coordinated, holistic resources—including interventions that address food insecurity and nutrition as central components of health.

## Food is Medicine: a model for syndemic care

Bridging the gap between social determinants of health and clinical practice, Food is Medicine (FIM) interventions offer a promising model for operationalizing syndemic care for prevention and treatment. FIM initiatives—a spectrum of food-based, healthcare-integrated interventions that often provide specific types of food and nutrition education and counseling—have gained traction as viable mechanisms for simultaneously improving food security and health outcomes (25). With flexible service options, including medically tailored meals, medically tailored groceries, and produce prescriptions, FIM supports individuals across the lifecourse, from prenatal programs to those for older adults with diet-related conditions, offering a continuum of care that spans prevention through treatment. In addition to addressing food insecurity, FIM interventions are increasingly recognized for their potential to advance health equity, with emerging evidence of positive effects on mental health and overall wellbeing. Systematic reviews and meta-analyses indicate that FIM interventions can improve food security, dietary patterns, and physical health outcomes for patients with diverse chronic conditions, including type 2 diabetes, cardiovascular diseases, HIV, and cancer (25, 26). Focusing on proximate mechanisms, Dixon and Mendenhall identify stress-mediation as a core component of interventions adopting a syndemic care lens (24).

Despite promising early results, many FIM studies have focused on short-term physical health outcomes such as body mass index, HbA1c, and blood pressure. Far fewer have examined effects on mental health or psychosocial stress—key elements of the syndemic framework. However, a growing number of mixed method and qualitative studies of FIM participants have documented changes in psychosocial stress, anxiety, depression, and loneliness (27–30). For example, a randomized trial involving 191 patients with HIV, participants who received 6 months of medically tailored meals had 68% lower odds of experiencing depressive symptoms compared to controls (31). Thus, current evidence demonstrates the significant and multifaceted health consequences of food and nutrition insecurity, positions mental health and psychosocial stress as part of a pathway underlying insecurity and chronic cardiometabolic conditions and shows the promise of FIM as a therapeutic tool for improving wellbeing.

Building on the syndemic framework, FIM interventions may act as critical stress-mediating strategies, aligned with Dixon and Mendenhall’s proposal of syndemic care. Current evidence suggests that FIM interventions can alleviate food and nutrition insecurity, concurrently reducing stressors by improving access to healthy foods, fostering social support, and enhancing self-efficacy in disease management. Biologically, these interventions may disrupt the interconnected physiological pathways whereby chronic stress triggers cortisol dysregulation and systemic inflammation, which in turn exacerbates cardiometabolic disease progression and management.

Figure 1 illustrates the intersecting biological, behavioral, and structural pathways through which food insecurity contributes to syndemic disease—and how interventions like FIM can disrupt these feedback loops. As depicted in Figure 1, FIM can break biological-social linkages by addressing social determinants like economic hardship and limited food access that “get under the skin” to influence mental and physical health. By targeting these intersecting biological and social pathways, FIM serves not only to improve diet and health outcomes but also to potentially disrupt the bidirectional feedback loops that perpetuate syndemic cycles of disadvantage and ill health. Yet, documented experiences of patients receiving FIM often reveal co-occurring social determinants of health, such as housing instability, unreliable transportation, and economic burdens that limit the long-term impact of FIM interventions alone (32). This underscores the importance of considering the broader social and structural conditions in which FIM efforts are embedded.

## Reconsidering the social and structural conditions

As promising evidence grows for FIM as a strategy aligned with syndemic care, it becomes increasingly important to situate these interventions within the structural and policy contexts that shape their implementation, effectiveness, and long-term potential. Current funding for FIM initiatives is heterogeneous, encompassing state-specific Medicaid demonstration programs, in lieu of services benefits, select Medicare Advantage plans, and philanthropic support for both research and implementation (33). To date, most interventions are time-limited, ranging from a few weeks to a year or more, but offer demonstrated short-term benefits for health (25). These benefits may also include reduced economic burden of food purchasing, enabling household resources to be saved or spent on other resources. In addition, interventions that provide nutrition and/or culinary education in tandem with provision of food may foster skills related to resource budgeting that may be sustained post-intervention.

Growing evidence from clinical trials and simulation studies suggests that FIM interventions may also be cost-effective for healthcare by reducing utilization and healthcare expenditures. For example, a recent simulation study estimated that medically tailored meal programs could save up to $13.6 billion annually in healthcare costs and prevent 1.6 million hospitalizations among Medicare and Medicaid beneficiaries (34). These findings suggest that the upfront investment in FIM may be offset by downstream savings in healthcare.

However, the sustainability of these improvements may be challenged by persistent economic and environmental barriers, as highlighted in select post-intervention studies, and with shifts in federal and state-level policies related to healthcare and food access (35). While FIM focuses on proximal factors, such as immediate access to nutritious foods, it often does not address the root causes—disparities in income, food environments, discrimination, and opportunity—that drive food insecurity and many health disparities (7, 36). This underscores the opportunity to explore how FIM interventions might be complemented by policies and structural strategies that target these ultimate, root causes. As enthusiasm for FIM grows, it is critical to examine the broader economic, structural, and policy conditions that shape its implementation and sustainability. Without such context, even promising interventions may fall short of achieving their full impact.

Gaps remain in understanding the long-term impacts and sustainability of FIM, especially across diverse and structurally disadvantaged communities. Advancing this work could involve integrating FIM into longer-term health and social care models, aligning with upstream initiatives to improve food environments and economic security. As the field evolves, attention to the contextual conditions that support or hinder the durability of FIM’s impact will be essential to fully realizing its potential as part of a more equitable approach to healthcare.

Critically, efforts to expand reach and impact of FIM risk being undermined by proposed federal policy changes that directly impact foundational funding and access mechanisms supporting FIM. For example, the proposed *One Big Beautiful Bill Act* includes provisions that would significantly reduce coverage under Medicaid and the Supplemental Nutrition Assistance Program (SNAP). Proposed Medicaid reductions are estimated to result in the loss of health coverage for millions of Americans. These cuts are further compounded by stringent work requirements and more restrictive eligibility criteria, which will make it more challenging to enroll in, and maintain, Medicaid coverage. Given that many FIM initiatives are reimbursed or piloted through Medicaid Section 1115 waivers or managed care organizations, diminished enrollment may translate to reduced program reach and weaker integration between screening and referral within clinical workflows.

In parallel, proposed changes to SNAP coverage, including tightened eligibility thresholds and procedural barriers, are likely to reduce participation. These reductions in access may increase food insecurity and threaten a fundamental food safety-net that currently supports over 40 million people. These policy shifts jeopardize not only the future of FIM, but also the broader landscape of syndemic care models across the United States.

## Syndemic solutions: implications for practice and policy

Syndemic theory has the potential to illuminate the ways in which human health is affected by structural and proximate factors that shape our environments and lived experiences, including those with food. As FIM interventions expand, integrating a syndemic lens could enhance efforts to address nutrition and the underlying structural and social contexts in which health inequities develop and persist. Ultimately, addressing syndemics requires collaboration among diverse sectors, including housing, social services, education, urban planning, and food system stakeholders whose policies collectively shape social determinants of health. In the context of shifting federal policies and funding, FIM practitioners may pursue pragmatic, locally grounded strategies that remain viable in the absence federal policy levers. For example, clinics and FIM practitioners might prioritize adapting existing technological infrastructure, such as electronic health records and data sharing systems, to strengthen shared referral platforms and improve care coordination with wraparound services. Technology can also support more holistic lifestyle-based approaches in FIM, as demonstrated by efforts to develop AI-driven smartphone applications (37). Aligning FIM programming with local food access and public health initiatives may enhance program stability by leveraging community-based resources, with braided funding streams serving as a mechanism to meet programmatic needs across sectors. Such integration can incorporate pre-existing efforts such as community gardens, nutrition education, and counseling. Integrated models that link clinical care with social interventions and community development hold promise for disrupting entrenched cycles of disadvantage and poor health. Likewise, engaging youth and younger adults can strengthen efforts toward disease prevention alongside management and treatment. Given nutrition’s profound influence on both biological processes and social determinants, centering food and nutrition security within syndemic-informed interventions is essential to advancing health equity and transforming the root conditions that sustain chronic disease burdens.

## Data Availability

The original contributions presented in the study are included in the article/supplementary material, further inquiries can be directed to the corresponding author.

## References

[1] Watson KB Wiltz JL Nhim K Kaufmann RB Thomas CW Greenlund KJ. Trends in multiple chronic conditions among US adults, by life stage, behavioral risk factor surveillance system, 2013–2023. Prev Chronic Dis. (2025) 22:22. doi: 10.5888/pcd22.240539, 40245168 PMC12007472

[2] Shaw KM Theis KA Self-Brown S Roblin DW Barker L. Chronic disease disparities by county economic status and metropolitan classification, behavioral risk factor surveillance system, 2013. Prev Chronic Dis. (2016) 13:160088. doi: 10.5888/pcd13.160088, 27584875 PMC5008860

[3] Satia JA. Diet-related disparities: understanding the problem and accelerating solutions. J Am Diet Assoc. (2009) 109:610–5. doi: 10.1016/j.jada.2008.12.019, 19328255 PMC2729116

[4] Bailey ZD Krieger N Agénor M Graves J Linos N Bassett MT. Structural racism and health inequities in the USA: evidence and interventions. Lancet. (2017) 389:1453–63. doi: 10.1016/S0140-6736(17)30569-X, 28402827

[5] Gundersen C Ziliak JP. Food insecurity and health outcomes. Health Aff. (2015) 34:1830–9. doi: 10.1377/hlthaff.2015.0645, 26526240

[6] Parekh T Xue H Cheskin LJ Cuellar AE. Food insecurity and housing instability as determinants of cardiovascular health outcomes: a systematic review. Nutr Metab Cardiovasc Dis. (2022) 32:1590–608. doi: 10.1016/j.numecd.2022.03.025, 35487828

[7] Odoms-Young A Bruce MA. Examining the impact of structural racism on food insecurity implications for addressing racial/ethnic disparities. Fam Community Health. (2018) 41:S3–6. doi: 10.1097/FCH.0000000000000183, 29461310 PMC5823283

[8] Rabbitt MP Reed-Jones M Hales LJ Suttles S Burke MP. Household food security in the United States in 2024 (2025). Available online at: www.ers.usda.gov (accessed December 22, 2025).

[9] Rabitt MP Jales LJ Reed-Jones M. Food security in the U.S. - key statistics & graphics. Washington, DC: USDA Economic Research Service (2025).

[10] Mahase E. Trump’s “big beautiful bill” could lead to 16 000 extra deaths a year, say researchers. BMJ. (2025) 389:r1250. doi: 10.1136/bmj.r1250, 40527547

[11] Seligman HK Bindman AB Vittinghoff E Kanaya AM Kushel MB. Food insecurity is associated with diabetes mellitus: results from the National Health Examination and nutrition examination survey (NHANES) 1999-2002. J Gen Intern Med. (2007) 22:1018–23. doi: 10.1007/s11606-007-0192-6, 17436030 PMC2583797

[12] Seligman HK Tschann J Jacobs EA Fernandez A López A. Food insecurity and glycemic control among low-income patients with type 2 diabetes. Diabetes Care. (2012) 35:233–8. doi: 10.2337/dc11-1627, 22210570 PMC3263865

[13] Abdurahman AA Chaka EE Nedjat S Dorosty AR Majdzadeh R. The association of household food insecurity with the risk of type 2 diabetes mellitus in adults: a systematic review and meta-analysis. Eur J Nutr. (2019) 58:1341–50. doi: 10.1007/s00394-018-1705-2, 29721679

[14] Pourmotabbed A Moradi S Babaei A Ghavami A Mohammadi H Jalili C . Food insecurity and mental health: a systematic review and meta-analysis. Public Health Nutr. (2020) 23:1778–90. doi: 10.1017/S136898001900435X, 32174292 PMC10200655

[15] Holt RIG De Groot M Golden SH. Diabetes and depression. Curr Diab Rep. (2014) 14:491. doi: 10.1007/s11892-014-0491-3, 24743941 PMC4476048

[16] Mendenhall E Kohrt BA Norris SA Ndetei D Prabhakaran D. Non-communicable disease syndemics: poverty, depression, and diabetes among low-income populations. Lancet. (2017) 389:951–63. doi: 10.1016/S0140-6736(17)30402-6, 28271846 PMC5491333

[17] Himmelgreen D Romero-Daza N Heuer J Lucas W Salinas-Miranda AA Stoddard T. Using syndemic theory to understand food insecurity and diet-related chronic diseases. Soc Sci Med. (2022) 295:295. doi: 10.1016/j.socscimed.2020.113124, 32586635

[18] Singer M Bulled N Ostrach B Mendenhall E. Syndemics and the biosocial conception of health. Lancet. (2017) 389:941–50. doi: 10.1016/S0140-6736(17)30003-X, 28271845

[19] Mendenhall E. Beyond comorbidity: a critical perspective of syndemic depression and diabetes in cross-cultural contexts. Med Anthropol Q. (2016) 30:462–78. doi: 10.1111/maq.12215, 25865829 PMC4600415

[20] Gowda C Hadley C Aiello AE. The association between food insecurity and inflammation in the US adult population. Am J Public Health. (2012) 102:1579–86. doi: 10.2105/AJPH.2011.300551, 22698057 PMC3464824

[21] Bermúdez-Millán A Wagner JA Feinn RS Segura-Pérez S Damio G Chhabra J . Inflammation and stress biomarkers mediate the association between household food insecurity and insulin resistance among Latinos with type 2 diabetes. J Nutr. (2019) 149:982–8. doi: 10.1093/jn/nxz021, 31006809 PMC6543200

[22] Hadley C Crooks DL. Coping and the biosocial consequences of food insecurity in the 21st century. Yearb Phys Anthropol. (2012) 149 Suppl 55:72–94. doi: 10.1002/ajpa.22161, 23109261

[23] Berkowitz SA Seligman HK Mozaffarian D. A new approach to guide research and policy at the intersection of income, food, nutrition, and health. Health Aff. (2025) 44:384–90. doi: 10.1377/hlthaff.2024.01346, 40193831 PMC12178120

[24] Dixon J Mendenhall E. Syndemic thinking to address multimorbidity and its structural determinants. Nat Rev Dis Primers Nat Res. (2023) 9. doi: 10.1038/s41572-023-00437-2 37142626

[25] Mozaffarian D Aspry KE Garfield K Kris-Etherton P Seligman H Velarde GP . “Food is medicine” strategies for nutrition security and cardiometabolic health equity: JACC state-of-the-art review. J Am Coll Cardiol. (2024) 83:843–64. doi: 10.1016/j.jacc.2023.12.023, 38383100

[26] Bhat S Coyle DH Trieu K Neal B Mozaffarian D Marklund M . Healthy food prescription programs and their impact on dietary behavior and cardiometabolic risk factors: a systematic review and meta-analysis. Adv Nutr. (2021) 12:1944–56. doi: 10.1093/advances/nmab039, 33999108 PMC8483962

[27] Thompson-Lastad A Chiu DT Ruvalcaba D Chen WT Tester J Xiao L . Food as medicine, community as medicine: mental health effects of a social care intervention. Health Serv Res. (2025) 60 Suppl 3:e14431. doi: 10.1111/1475-6773.14431, 39775914 PMC12052511

[28] Berkowitz SA Shahid NN Terranova J Steiner B Ruazol MP Singh R . “I was able to eat what i am supposed to eat” - patient reflections on a medically-tailored meal intervention: a qualitative analysis. BMC Endocr Disord. (2020) 20. doi: 10.1186/s12902-020-0491-z, 31959176 PMC6971854

[29] RadtkeMD TesterJM XiaoL ChenW Emmert-AronsonBO MarkleEA . Impact of a multicomponent food-as-medicine intervention on behavioral and mental health outcomes for patients with and without food insecurity. Nutrition. (2025) 134:112734. doi: 10.1016/j.nut.2025.11273440132449

[30] Heuer JN Romero-Daza N Gray D Lehigh G Webb WA Himmelgreen D. A qualitative evaluation of a food prescription program during the COVID-19 pandemic. Ann Anthropol Pract. (2025) 49:e70025. doi: 10.1111/napa.70025

[31] Palar K Sheira LA Frongillo EA O’Donnell AA Nápoles TM Ryle M . Food is medicine for human immunodeficiency virus: improved health and hospitalizations in the changing health through food support (CHEFS-HIV) pragmatic randomized trial. J Infect Dis. (2024) 231:573–582. doi: 10.1093/infdis/jiae195, 38696724 PMC11911788

[32] Himmelgreen D Romero-Daza N Webb WA Heuer JN Gray D Lehigh GR. Implementing a food prescription program during COVID-19: benefits and barriers. Healthcare. (2024) 12:182. doi: 10.3390/healthcare12020182, 38255070 PMC10815315

[33] Hanson E Albert-Rozenberg D Garfield KM Leib EB Ridberg RA Hager K . The evolution and scope of Medicaid section 1115 demonstrations to address nutrition: a US survey. Health Affairs Scholar. (2024) 2:1–9. doi: 10.1093/haschl/qxae013, 38577164 PMC10986195

[34] Hager K Cudhea FP Wong JB Berkowitz SA Downer S Lauren BN . Association of national expansion of insurance coverage of medically tailored meals with estimated hospitalizations and health care expenditures in the US. JAMA Netw Open. (2022) 5:E2236898. doi: 10.1001/jamanetworkopen.2022.36898, 36251292 PMC9577678

[35] Schlosser AV Joshi K Smith S Thornton A Bolen SD Trapl ES. “The coupons and stuff just made it possible”: economic constraints and patient experiences of a produce prescription program. Transl Behav Med. (2019) 9:875–83. doi: 10.1093/tbm/ibz086, 31570919 PMC6937548

[36] Drewnowski A. Food insecurity has economic root causes. Nat Food. (2022) 3:555–6. doi: 10.1038/s43016-022-00577-w, 35965676 PMC9362113

[37] DeBate RD Templeton JM Hiteshkumar Jariwala J Bleck J. *MyFoodRx*: formative research for the development of an AI-based personalized food-as-medicine smartphone application. Am J Lifestyle Med. (2026) 1–14. doi: 10.1177/15598276261417226 PMC12812061 41555970

